# Aldehyde Dehydrogenase 3 Is an Expanded Gene Family with Potential Adaptive Roles in Chickpea

**DOI:** 10.3390/plants10112429

**Published:** 2021-11-10

**Authors:** Rocío Carmona-Molero, Jose C. Jimenez-Lopez, Cristina Caballo, Juan Gil, Teresa Millán, Jose V. Die

**Affiliations:** 1 Department of Genetics ETSIAM, University of Córdoba, 14071 Córdoba, Spain; z72camor@uco.es (R.C.-M.); juan.gil@uco.es (J.G.); teresa.millan@uco.es (T.M.); 2 Department of Biochemistry, Cell and Molecular Biology of Plants, EEZ-CSIC, 18008 Granada, Spain; josecarlos.jimenez@eez.csic.es; 3 Institute of Agriculture and School of Agriculture and Environment, The University of Western Australia, Perth 6009, Australia; 4 Área de Genómica y Biotecnología, IFAPA, Alameda del Obispo, 14080 Córdoba, Spain; cristinacaballolinares@gmail.com

**Keywords:** abiotic stress, ALDH, chickpea, EST, legumes, *Fusarium*, oxidative stress, qPCR, SRA

## Abstract

Legumes play an important role in ensuring food security, improving nutrition and enhancing ecosystem resilience. Chickpea is a globally important grain legume adapted to semi-arid regions under rain-fed conditions. A growing body of research shows that aldehyde dehydrogenases (ALDHs) represent a gene class with promising potential for plant adaptation improvement. Aldehyde dehydrogenases constitute a superfamily of proteins with important functions as ‘aldehyde scavengers’ by detoxifying aldehydes molecules, and thus play important roles in stress responses. We performed a comprehensive study of the ALDH superfamily in the chickpea genome and identified 27 unique ALDH *loci*. Most chickpea ALDHs originated from duplication events and the ALDH3 gene family was noticeably expanded. Based on the physical locations of genes and sequence similarities, our results suggest that segmental duplication is a major driving force in the expansion of the ALDH family. Supported by expression data, the findings of this study offer new potential target genes for improving stress tolerance in chickpea that will be useful for breeding programs.

## 1. Introduction

Aldehyde molecules are common intermediates of a number of catabolic and biosynthetic pathways that are produced in response to biotic and abiotic environmental stresses. Although aldehydes are indispensable to developmental and growth processes, excessive amounts of aldehydes interfere with metabolism, becoming toxic, so their unbalanced levels must be regulated within the cells [1,2]. The aldehyde dehydrogenase (ALDH) superfamily is a group of NAD(P)+-dependent enzymes that catalyze the irreversible oxidation of a wide range of reactive aldehydes to their corresponding carboxylic acids [3,4]. In addition, under conditions inducing oxidative stress, ALDH enzymes act as ‘aldehyde scavengers’ by metabolizing reactive aldehydes derived as lipid peroxidation-derived aldehydes, which are potentially toxic due to their extreme reactivity with the nucleophilic compounds such as nucleic acids, proteins and membrane lipids [5,6]. However, ALDH activity may also serve to fine-tune gene activation since ALDHs may modulate signaling by lipid peroxidation-derived bioactive aldehydes [7].

Interestingly, ALDHs are found throughout all taxa including both prokaryotes and eukaryotes, where many ALDH families are highly conserved among animals and plants [8]. To date, ALDHs have been identified and categorized into 24 separate families based on protein sequence identity as main criteria [9], but also by their functionality [10,11].

The first identified plant ALDH gene *rf2,* which encodes a mitochondrial class-2 ALDH, was reported to function as a male fertility restorer (RF) protein of maize [12]. Since then, many other ALDH were classified as RF afterward [13], and a number of studies demonstrated that ALDH genes are involved in diverse pathways with crucial roles in molecular detoxification, as well as growth and development [14,15,16]. In addition, many of the plant ALDH genes characterized to date are induced under a wide range of abiotic stresses such as drought, cold, high salinity and heavy metals, highlighting their potential role in improving stress tolerance/environmental adaptation [2,17,18,19].

The identification of ALDH genes in different crop species has soared in recent times due to the increasing numbers of plant species that have been sequenced. Among the plant species containing 14 distinct ALDH families, the ALDH11, 12, 19, 21, 22, 23 and 24 are unique in the Plantae kingdom. The single gene of the ALDH19 family reported so far encodes a gamma-glutamyl phosphate reductase involved in proline biosynthesis [20]; no other higher plant has been found to contain this family. 

Chickpea (*Cicer arietinum* L.) is globally the second most important grain legume [21]. Although its yield potential has increased in recent years, its global production is constrained by several major abiotic (drought, heat, high salinity) and biotic stressors such as the fungal diseases Fusarium wilt, and Ascochyta blight, which may cause 100% loss in yield when conditions are favorable for infection [22,23]. Until recently, lack of information on legume genomes traditionally restricted the survey of gene functionalities in response to the environment or stress, which may be valuable for implementation in breeding programs for chickpea yield improvements under climate change immediate adaptation. Fortunately, the genome sequence of chickpea has become available in the last few years, providing an unprecedented resource that can be exploited in numerous ways [24,25].

In the present study, we identified 27 ALDH *loci* in the chickpea genome encoding a total number of 45 proteins that contained the complete ALDH domain and belonged to 10 different ALDH families. We performed a comprehensive functional comparison of the chickpea ALDH superfamily to other sequenced plant species, through phylogenetic and synteny analyses, and the study of their expression profiles in response to various types of stress. Results from this study provide functional targets with yield improvement potential for chickpea breeding programs, as well as the basis for further comparative genomic analysis and a framework to study the ALDH genes’ evolution on a large timescale within the legume family. 

## 2. Results and Discussion

### 2.1. ALDH Gene Family in the Chickpea Genome

**Figure 1:**
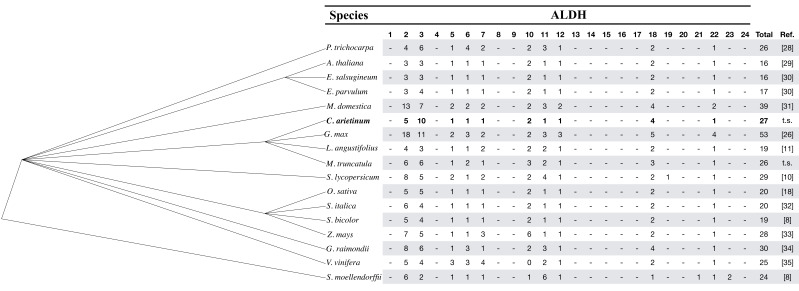
Distribution of ALDH families (1–24) in several species: the phylogenetic tree on the left, based on the taxonomic identifications of the species, was generated using the Taxonomy Common Tree Tools on the NCBI website (http://www.ncbi.nlm.nih.gov/guide/taxonomy/, accessed on 20 November 2019). The names of the ALDH families are listed on top of the table. The references (Ref.) are as follows: Brocker et al., 2013 [8]; Jimenez-Lopez et al., 2016 [10]; Jimenez-Lopez 2016 [11]; Gao et al., 2009 [18]; Wang et al., 2017 [26]; Tian et al., 2015 [28]; Kirch et al., 2004 [29]; Hou et al., 2015 [30]; Li et al., 2013 [31]; Chen et al., 2014 [32]; Zhou et al., 2012 [33]; He et al., 2014 [34]; Zhang et al., 2012 [35]; t.s. this study. Symbols − represent absence.

We identified 27 unique ALDH gene sequences from the chickpea genome through database and bioinformatics searches. Information on the 27 chickpea sequences (name, locus ID, length, location on chromosome and features about the deduced peptide) is listed in Table 1. The exon number of the CaALDH genes ranged from 5 (NCBI: LOC101502106) to 21 (LOC101490622 and LOC101512568). The sizes of the deduced proteins varied markedly from 134 (LOC101502106) to 759 (LOC101512568) amino acids. The corresponding molecular weights varied from 15.07 to 82.38 kDa and the predicted isoelectric points (pIs) varied broadly from 4.34 to 9.49. As exhibited in other plant species, the wide range of pIs suggests that the chickpea ALDH proteins can work in various different subcellular environments, which is in accordance with the subcellular localization predicted for the sequences revealing that 44.4% (12 out 27) of CaALDHs can be localized to the cytoplasm (Table S1). All 27 ALDH proteins contain a conserved ALDH domain (Pfam: PF00171) with variable length, which is a basic feature of ALDH families. The classification of protein families was made according to the criteria established by the ALDH Gene Nomenclature Committee (AGNC), namely the protein root symbol (ALDH) was followed by a family description number (1, 2, 3, etc.), a subfamily descriptor (A, B, C, etc.) and an individual gene number. As we used one gene model per *locus*, we did not include an extra lowercase letter to designate the number of variants. Thus, using the AGCN criteria, the ALDH proteins from chickpea fall into 10 families based on their sequence identities (Figure 1). These families are also present in other vascular plants, suggesting that these 10 families may have evolved before the divergence of magnoliophyta and pteridophyta. Six chickpea families are represented by a single gene (ALDH5, ALDH6, ALDH7, ALDH11, ALDH12 and ALDH22), whereas the remaining four families contain multiple members (ALDH2, ALDH3, ALDH10 and ALDH18). Families ALDH5, 12 and 22 are also defined by a single gene in *Arabidopsis* as well as some other plant species. It has been proposed that these families represent constitute housekeeping ALDH genes, involved in preservation of nontoxic aldehyde levels and central plant metabolism [10]. The ALDH2 family, which is the largest ALDH family in plants, contains five genes in chickpea. The ALDH3 family in chickpea is comparatively abundant, containing the largest number of members (10 genes) described in plants to date with the exception of soybean, whose expansion of the ALDH gene superfamily is mostly driven by whole-genome duplication events [26]. Thus, chickpea ALDH3 family may be functionally important in carrying out additional stresses-response proteins among ALDHs, enabling it to tolerate environmental stress such as salinity and drought through detoxification of molecules generated under these different stresses to maintain oxidative homeostasis. Four out of the fourteen distinct ALDH families seem to be missing in the chickpea genome (ALDH19, ALDH21, ALDH23 and ALDH24). It has been proposed that families ALDH21, ALDH23 and ALDH24 play important roles in the transition of aquatic plants to terrestrial plants. Then, these families were lost during the evolution of flowering plants [26,27]. The family ALDH19 is unique among plants as only a single gene has been found in tomato, suggesting that this gene played an important role during evolution of that species [10]. This gene encodes a γ-glutamyl phosphate reductase, which catalyzes the reduction of l-glutamate 5-phosphate to 1-glutamate 5-semialdehyde (NADP-dependent) during the biosynthesis of proline from glutamate [20].

Compared to other well-characterized plant ALDH families, such as *Arabidopsis*, grape or rice, chickpea contains one of the most expanded ones, following the 53 ALDH genes in *Glycine max*, 39 in *M. domestica*, 30 in *Gossypium raimondii*, 29 in *Solanum lycopersicum* and 28 in *Z. mays.* Similar to *Gossypium* spp. [14,34], or *Oryza sativa* [13], the four sequences of the chickpea ALDH18 family contain an AA-kinase domain, which is not found in other families, and lack the two other conserved sites (PS00687 and PS00070; Table S1).

**Figure 2:**
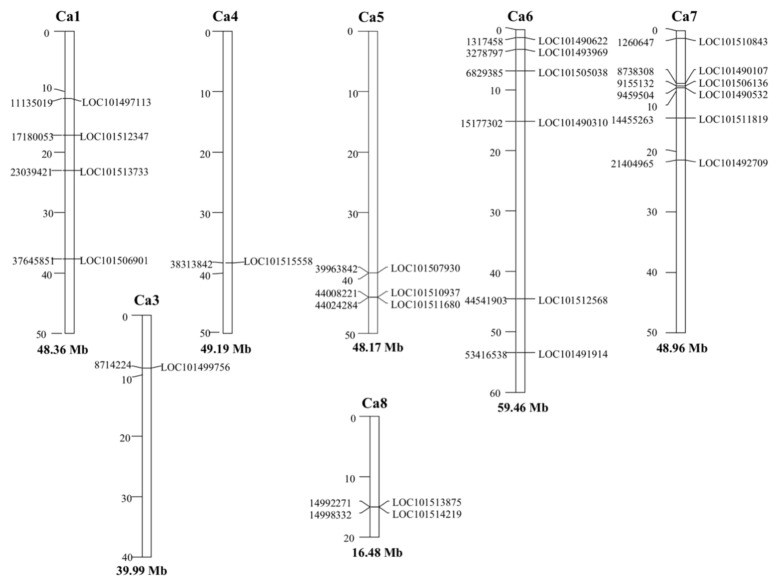
Genomic distribution of ALDH genes on chickpea chromosomes. Only those chromosomes bearing CaALDH genes are represented. The chromosome numbers and sizes (Mb) are indicated at the top and bottom of each bar, respectively.

In order to gain an insight into the genome organization, we mapped ALDH genes into chromosomes Based on the available *C. arietinum* genome assembly, 23 out of the 27 CaALDH genes were distributed among seven of the eight chromosomes. We could not map LOC101502106, LOC101497514, LOC101488602 (members of ALDH3 subfamily) and LOC105852801 (ALDH18B4). The other 23 ALDH genes were unevenly distributed through the chickpea genome. Two chromosomes contained the highest number with six ALDH genes (chromosome 6 and 7), whereas chromosome 3 and 4 contained one ALDH gene, respectively. Chromosome 8, which is the shortest in the chickpea genome, contained two ALDH genes (LOC101513875 and LOC101514219). No ALDH gene could be found in chromosome 2 (Figure 2).

### 2.2. Evolutionary Relationships of ALDH Gene Families between Chickpea and Medicago

In order to explore the evolution of the CaALDH genes, we compared the syntenic blocks of the chickpea and the model legume *Medicago truncatula* genomes. In previous studies, synteny analyses have revealed extensive conservation and good collinearity between both legumes [36,37]. In the current study, we identified large-scale syntenic blocks containing orthologues from six ALDH families (ALDH6, ALDH7, ALDH11, ALDH12, ALDH18 and ALDH22), including eight CaALDH genes from chickpea and eight ALDH genes from *Medicago* (Table S2). Five pairs of orthologous groups appeared to be single chickpea-to-*Medicago* ALDH gene correspondences. It is likely that these genes/families derived from a common ancestor of chickpea and *Medicago* conserved during evolution. Furthermore, we also found instances of a single chickpea gene corresponding to multiple *Medicago* genes, in addition to several chickpea duplications corresponding to a single *Medicago* gene. The remaining four chickpea families (ALDH2, ALDH3, ALDH5 and ALDH10) could not be mapped to any syntenic block.

### 2.3. Phylogenetic Analysis of Chickpea ALDH Genes

**Figure 3:**
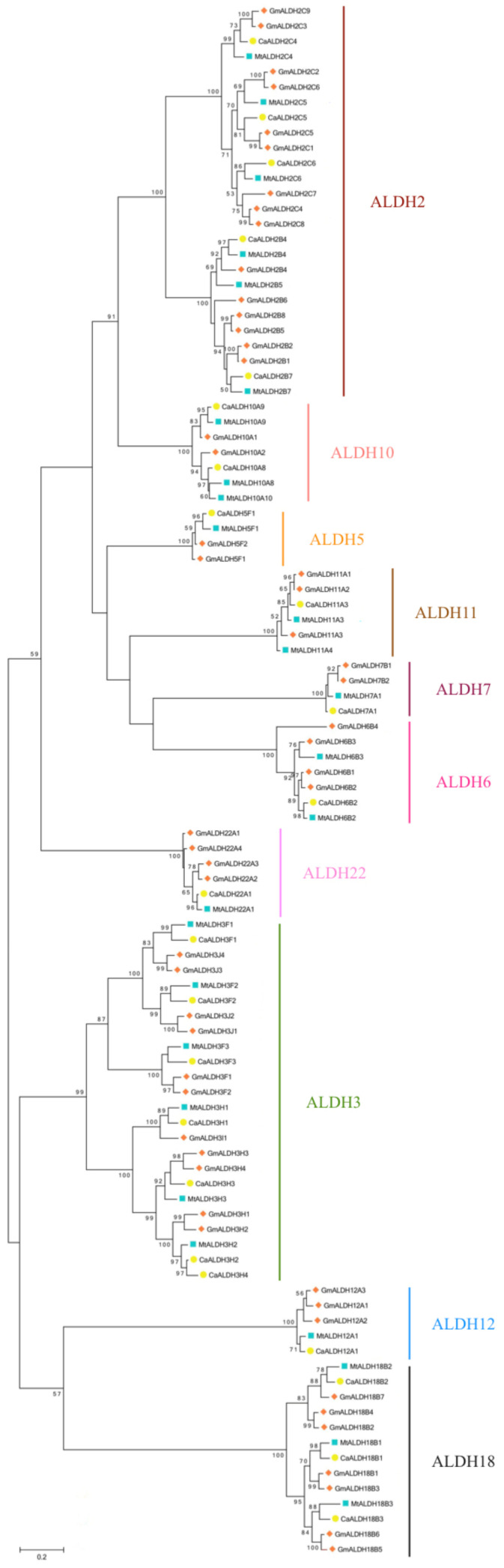
Phylogenetic tree of ALDH proteins from chickpea (Ca), *G. max* (Gm) and *M. truncatula* (Mt). Alignment of 102 ALDH protein sequences from four plant species was conducted with MUSCLE, and the phylogenetic tree was constructed using MEGA 6 based on the Maximum Likelihood method. Bootstrap values in percentage (1000 replicates) are labelled on the nodes. CaALDHs are marked with solid orange squares. Scale bar represents the number of substitutions per site.

To study the evolutionary relationship of the ALDH gene superfamily among different species, a phylogenetic tree was generated with a full-length of 102 well-characterized ALDH proteins from *G. max* and *M. truncatula* (Figure 3). This result was consistent with previous findings [14,32,38], and showed that different family proteins in the same species did not cluster together. However, it grouped the same family proteins of different species. The ALDH19 family is not included here, as our analyses did not incorporate any sequences from tomato [31]. The phylogenetic tree indicates that most of the ALDH families represent a common plant ALDH core (ALDH5, ALDH6, ALDH7, ALDH10, ALDH11, ALDH12, ALDH13, ALDH18, ALDH22). The ALDH18 family is the most phylogenetically distant group related to the remaining families, indicating that these proteins have the greatest degree of sequence divergence from the other ALDH families and do not contain the conserved ALDH active sites [8]. It is worth mentioning that the majority of CaALDHs grouped more closely to *M. truncatula* than to soybean, which is consistent with the evolutionary relationships among the three species. That was particularly clear with families ALDH5, ALDH12 and ALDH22, all of them represented by only one chickpea sequence, one *Medicago* sequence and several soybean sequences (Figure 1). The soybean genome most likely increased these ALDH families by duplication events, which seem to have greatly expanded all the ALDH families with the exception of ALDH10. It is noteworthy that the cluster with family ALDH3 was mostly made because of the remarkable expansion of this family in the chickpea genome. In *Arabidopsis*, the expression of class3 ALDHs is induced by environmental stresses such as drought, salinity, ABA exposure, heavy metals and pesticides [19,39,40,41]. The notable expansion of the CaALDH3 gene families compared with other plant species suggests that these ALDH genes may be essential for chickpea to cope with environmental stresses.

### 2.4. ALDH Expansion: Gene Duplications

The expansion of gene families is based on gene duplications, which in turn, mainly rely on segmental and tandem duplications [42]. Based on a comprehensive analysis of chromosomal locations and sequence similarities, 59.3% in 16 out of 27 ALDH sequences, ALDH genes appear to be associated with either local duplication events or duplications to unlinked *loci* (Figure S1). There is no support for tandemly duplicated ALDH genes in the genome of Chinese cabbage [38]; however, tandem duplications have been shown to occur in the ALDH family of grapes, apples and soybeans [26,31,35], as well as the monocot species rice and millet [18,32]. Chickpea ALDH genes mapped on the same chromosomes are candidates to have undergone local gene duplications. We found two genes on chromosome 5 (LOC101510937, LOC101511680) and two genes on chromosome 8 (LOC101513875, LOC101514219) that met the criteria to form a cluster as described in Section 3.2. These two pairs of genes are separated by <10 kb, respectively. The other duplicated genes (75% duplications) are located on different chromosomes, suggesting that segmental duplications play a major role in the expansion of the ALDH gene family in chickpea.

### 2.5. Expression Profiles of CaALDH Genes

In order to gain a more accurate insight into the functional roles of the ALDH genes, we analyzed their expression patterns in different tissues using available EST datasets [43]. Considering the stringent criterion described in Section 3.4, 11 ALDH genes had expression support (26 ESTs). One ALDH gene (LOC101506136) hit 8 ESTs, whereas LOC101510843 and LOC101490310 hit four and three ESTs, respectively (Table S3). Regarding the plant tissues, root tissue was the most common hit (18 hits) followed by leaves (5 hits). The experimental conditions of these libraries suggest an adaptative role in a variety of environmental responses by the ALDH superfamily. Most of the libraries were constructed in response to drought stress (14 hits) but we also found ESTs from libraries in responses to insect attack, Cd toxicity and response to thidiazuron, a synthetic plant regulator of morphogenetic processes that induces the expression of stress-related genes [44,45]. We also combined these data with publicly available RNA-seq analyses and confirmed the regulation of a number of chickpea ALDH genes as part of the transcriptional response in leaf tissues triggered by drought stress (Table S5; [46]).

**Figure 4:**
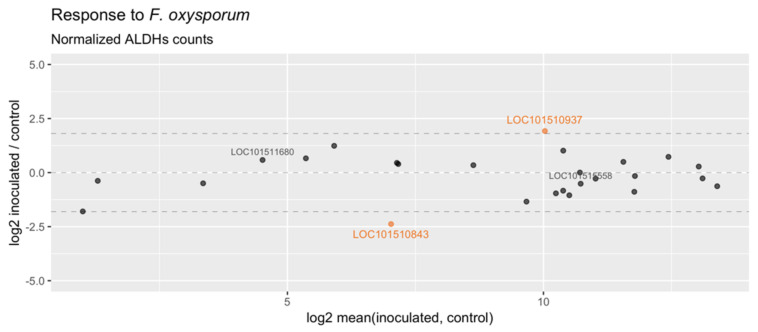
In silico expression analysis in response to *Fusarium oxysporum*: MA−plot of mean expression signal vs. log2−normalized counts of ALDH genes in two chickpea transcriptome libraries (inoculated vs. control). Genes highly enriched (counts ratio >3.5−fold) in any of the conditions are shown in orange color, whereas the duplicated genes LOC101511680 and LOC101515558 are shown in grey color.

Over the past decade, our laboratory has been working toward increasing the agronomic adaptation of chickpea on disease resistance. In particular, we are focused on delimiting the genomic regions that might help us to unveil the defense pathways during the interaction of the plant with the soil-borne fungus *Fusarium oxysporum*, which is a serious threat to chickpea production. Interestingly, five ESTs were found in specific subtracted cDNA libraries from infected roots with *Fusarium.* Based on this finding, we aimed to gain an insight into the role of the ALDHs in the response to the fungal disease. From publicly available transcriptome datasets, we selected two libraries constructed with the chickpea genotype WR315, as this genotype is commonly used as a resistant parental line in the breeding program. Some sequences showed extremely low ALDH count numbers, suggesting that they are expressed at very low levels in root tissues (LOC101497113, LOC101491914 and LOC101511819). Overall, the ALDH counts are highly correlated between non-inoculated and inoculated plants (R = 0.91). However, two genes were more abundant in a given condition: LOC101510843 (*CaALDH11A3*) was over-represented in roots of control plants, while LOC101510937 (*CaALDH3H2*) showed a larger count number in inoculated plants (Figure 4). Interestingly, LOC101515558 and LOC101511680 (*ALDH3H3* and *ALDH3H4*, respectively), which are duplicated with LOC101510937 (Figure S1), show a different expression pattern, as they are not differentially abundant in any condition. This result suggests that CaALDH3H2 and the duplicated sequences CaALDH3H3 and CaALDH3H4 are probably regulated in different ways. The remarkable expansion of the ALDH3 family in chickpea may have evolved as a consequence of functional specialization.

**Figure 5:**
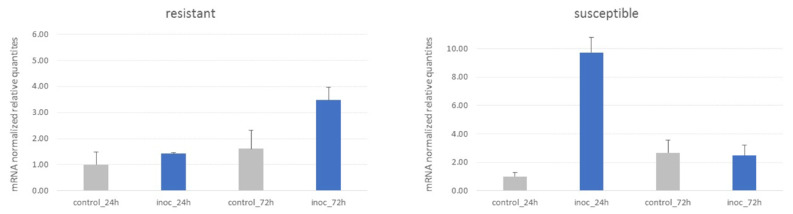
Gene expression levels of LOC101511819 in response to *Fusarium oxysporum*. Samples are a pair of NIL made of inoculated vs. control roots at 24 and 72 h post inoculation. Normalized relative quantities are rescaled to the control (24 h) sample. The data represent of two biological samples (mean ± SEM).

In our search for a deeper understanding of the ALDH role during the Fusarium wilt response, we further aimed at measuring the expression levels of representative candidates by RT-qPCR. We used a pair of near-isogenic lines (NILs) differing in their sensitivity to Fusarium race 5 (resistant vs. susceptible) to monitor the transcriptional changes in roots at 24 and 72 h post inoculation (hpi). NILs represent a powerful tool for improving our understanding of the molecular and genetic basis of agronomic traits as the pair of plants show nearly identical genetic background except for a single section/locus on a given chromosome, so that only a small target region of the genome segregated [47]. Seven out eight candidate genes that we tested did not exhibited regulation |2-fold change| in response to Fusarium wilt in any of the genotypes (Figure S2). In chickpea, Foc resistance has been reported to be race-specific [48] and subtle deviations from conserved signaling mechanisms may occur leading to specific plant–pathogen interactions, which in turn may explain the apparent lack of agreement between in silico and experimental data. Although no regulation of most of the ALDH candidates seems to be induced in our material, the LOC101511819 (*CaALDH3F3*) is clearly upregulated in infected roots of the susceptible and resistant NILs at 24 and 72 hpi, respectively (Figure 5). This is interesting because the appropriate timing of gene regulation that leads to Foc5 pathogen recognition has been suggested as a distinct feature of the NIL pair [49]. The encoded protein by LOC101511819 is highly conserved among other legumes and shares >80% identity at the amino acid level with the homologue of *M. truncatula*, *L. angustifolius*, *G. max*, *A. hypogaea* and *P. vulgaris*, among others. LOC101511819 is a particularly valid candidate for further experimental validation.

## 3. Materials and Methods

### 3.1. Database Searches and Annotation of ALDH Genes 

Comprehensive identification of *C. arietinum* ALDH gene family members was achieved using *Arabidopsis thaliana*, *Glycine max* and *Medicago truncatula* ALDH proteins. A keyword-based search was carried out against the databases of the National Center for Biotechnology Information (NCBI) to extract 136 *A. thaliana* and 55 *G. max* ALDHs. In addition, 36 *M. truncatula* ALDHs were downloaded from the Phytozome v12.1 database (https://phytozome.jgi.doe.gov, accessed on 10 April 2019). All these sequences were used as queries in BLASTP searches [50] to identify the corresponding ALDH members in the chickpea proteome using a cut-off of query coverage ≥25%, E-value ≥ 1 × 10^−25^, and identity ≥25%. The Pfam domain PF00171 (ALDH family), PS00070 (ALDH cysteine active site), PS00687 (ALDH glutamic acid active site), and the accession ‘cl11961’ were queried against the Pfam (https://pfam.xfam.org/, accessed on 10 April 2019) and the CDD (https://www.ncbi.nlm.nih.gov/cdd/, accessed on 10 April 2019) databases to confirm the candidate sequences as ALDH proteins. For exhaustive identification of divergent members, we used the chickpea sequences as queries in BLASTP searches against the chickpea proteome. These steps enabled us to obtain 45 unique ALDH protein sequences. Using one gene model per *locus*, we identified 27 *C. arietinum* non-redundant ALDH genes (CaALDH). Information on chromosomal location, locus ID, amino acid length, molecular weight and number of exons was retrieved from the NCBI using the *refseqR* package [51]. The ExPASy proteomics server database (https://www.expasy.org/, accessed on 10 April 2019) was used to predict the theoretical isoelectric point (pI) of each ALDH protein, as well as the molecular weights (MW) of the deduced proteins without that record in the NCBI. For subcellular localization predictions and active site assessment, we used DeepLoc 1.0 [52], SLP-Local [53], SMART, ChloroP 1.1 [54], Mitoprot [55], PROSITE and PROPSEARCH databases [56]. Putative ALDHs were further annotated on the basis of the ALDH Gene Nomenclature Committee (AGNC) annotation criteria [57]. Briefly, amino acid sequences that shared >40% identity to previously identified ALDH sequences were considered to comprise a family; those exhibiting >60% identity comprise a protein subfamily, while sequences with <40% identity are considered to be a new family. The ALDH sequences from *Medicago truncatula* shown in Figure 1 were annotated using the same method as chickpea.

### 3.2. Syntenic Blocks and Gene Duplication Analysis

Syntenic blocks between chickpea and *M. truncatula* genomes were downloaded from the Plant Genome Duplication Database [58]. Those containing CaALDH genes were identified and analyzed. Duplicated genes were labelled as ‘duplicated genes’ according to the criteria defined by [59]: (1) the alignment covered >70% of the longer gene; (2) the aligned region had an identity of >70%. Coverage and identity values were obtained by BLAST searches of all the predicted CDS against each other. Tandem duplicated genes were defined as those closely related in the same family and clustered together within a sliding window size <250 kb [60]. The Circoletto tool was used to plot sequence similarity [61]. 

### 3.3. Phylogenetic Analysis of ALDH Gene Families

To carry out the phylogenetic analysis, the alignments of the deduced amino acid ALDH protein sequences from *M. truncatula*, soybean and chickpea were performed using the MUSCLE program as implemented in the Molecular Evolutionary Genetics Analysis software (MEGA) version 6 with default options [62]. The alignments were created using the Gonnet protein weight matrix. Sequences < 250 aa were eliminated from the matrix because short sequences interfered with a fine alignment. Additionally, the AA-kinase domain contained by the ALDH18 family was eliminated from the alignment. A total of 102 proteins were finally used to build the ALDH phylogeny of chickpea. The phylogenetic tree was constructed using the Maximum Likelihood method implemented in MEGA and the reliability of the interior nodes was assessed using 1000 bootstrap replicates.

### 3.4. In Silico Expression Analysis

The coding sequences of ALDH genes were used to query the NCBI chickpea ESTs. Searching parameters were set as follows: blast algorithm megablast, identity > 95%, query coverage > 25% and E-values < 10^−20^. Next, the full-length CDS of the ALDH genes were employed to query the NCBI Sequence Read Archive (SRA) database (https://www.ncbi.nlm.nih.gov/sra, accessed on 10 April 2019). For assessment of ALDHs expression support in response to the fungus *Fusarium oxysporum*, we selected two libraries constructed from infected root samples of resistant (WR315) chickpea plants after 48 h post-inoculation (SRX535351), and control samples of resistant (WR315) chickpea plants (SRX535349) using Magic-BLAST, a novel tool allowing the mapping of large next-generation sequencing runs against a reference database [63]. The searching parameters were implemented as follows: only one read per hit was counted, length reads were equivalent to 100 bp, and the identity > 99%. Normalized counts of hits were performed using public scripts to quantify the expression of transcripts from datasets (https://github.com/NCBI-Hackathons/SimpleGeneExpression, accessed on 10 April 2019).

### 3.5. Plant Material and Pathogen Inoculation

Plant material and treatment have been described in detail elsewhere [49]. Briefly, a pair of near isogenic lines RIP8-94-5 resistant (R)/RIP8-94-11 susceptible (S)—segregant to *Fusarium oxysporum* race 5 resistance were grown in controlled conditions under a temperature regime of 25 and 22 °C and 12 h photoperiod under fluorescent light. Plants at the three to four node stages were inoculated with a concentration of spores adjusted to 1 × 106 spores ml-1 following the method described by [64]. Root samples were collected and pooled from at least 4 inoculated and non-inoculated plants at 24, and 72 h post-inoculation. Samples were frozen in liquid nitrogen immediately after harvesting and stored at −80 °C. Two biological repetitions per time-point were performed.

### 3.6. RNA Isolation, cDNA Synthesis and Quality Controls

Total RNA from all samples was isolated using the TRISURE reagent protocol (Bioline). RNA concentration was determined by measuring the optical density using a NanoDrop spectrophotometer with A260/A280 ratio between 1.9 and 2.1 and A260/A230 greater than 2.0. To avoid any genomic DNA (gDNA) contamination, ~10 μg of RNA extracts were treated with TURBO DNase I (Life Technologies) before cDNA synthesis. Complementary DNAs was synthesized by priming with oligodT_12–18_ (Life Technologies), using SuperScript III Reverse Transcriptase (Invitrogen) following the instructions of the provider. The cDNAs were diluted to a final volume of 20 μL. Then, we tested the presence of genomic DNA (gDNA) contamination in the cDNA samples using a primer pair designed in two different exons of the NAD-dependent malic chickpea sequence XM_004510782 [49]. To infer the integrity of the total RNA and assess the quality of the reverse transcriptase reaction, we used a 3′:5′ amplification ratio assessment [65]. This assay aimed at measuring the integrity of the NAD-dependent malic sequence (XM_004510782). For this assay, we used two primer pairs that amplify two cDNA fragments, one from the 5′ end (81 bp) and one from the 3′ region (80 bp) of the malic gene. The fragments are 1180 and 460 bp, respectively, from the 3′ end of the cDNA [49]. The 3′:5′ amplification ratio of the malic cDNA fragments was calculated using the comparative Cq method [66]. The average ratio was 1.18 ± 0.59 (mean, SD). All ratios were <1.5-fold and the cDNAs were judged to be suitable for qPCR analysis [67]. Reliability of the interior nodes was assessed using 1000 bootstrap replicates. 

### 3.7. Real-Time qPCR Assays

Primer sequences were designed to amplify 8 candidate genes based on the phylogenetic and in silico analyses. Primers were designed using the following criteria: Tm of 60 ± 1 °C and PCR amplicon lengths of 80–100 bp, yielding primer sequences with lengths of 19–23 nucleotides and GC contents of 40–80%. For predicting the secondary structure of the amplicons, we used MFOLD version 3.4 software with default settings of minimal free energy, 50 mM Na^+^, 3 mM Mg^2+^ and an annealing temperature of 60 °C [68]. We chose primers that would yield amplicons with minimal secondary structures and melting temperatures that would not hamper annealing. Designed primers were synthesized by Integrated DNA Technologies (Leuven, Belgium). Table S4 shows the primer sequence and the overall mean real-time PCR amplification efficiency of each primer pair (E) estimated from the data obtained from the exponential phase of each individual amplification plot and the equation (1 + E) = 10^slope^ using LinReg software and the criteria of including three–five fluorescent data points with R^2^ ≥ 0.998 to define a linear regression line [69].

PCR reactions were carried out in a CFX Connect Real-Time System thermal cycler (Bio-Rad, Hercules, CA, USA) using iTaq Universal SYBR Green Supermix (Bio-Rad) to monitor dsDNA synthesis. Reactions contained 1.5 μL of the diluted cDNA as a template and 0.2 μM of each primer in a total volume reaction of 10 μL. Master mix was prepared and dispensed into individual wells using electronic Eppendorf Xplorer^®^ multipipettes (Eppendorf AG, Germany). The following standard thermal profile was used for all PCRs: polymerase activation (95 °C for 3 min), amplification and quantification cycles repeated 40 times (95 °C for 3 s, 60 °C for 30 s). The specificity of the primer pairs was checked by melting-curve analysis performed by the PCR machine after 40 amplification cycles (60–95 °C). Fluorescence was analyzed using Bio-Rad CFX Manager analysis software v2.1. All amplification plots were analyzed using a baseline threshold of 75 relative fluorescence units (RFU) to obtain Cq (quantification cycle) values for each gene–cDNA combination. Calculations were performed using the advanced quantification model with efficiency correction, multiple reference genes normalization and use of error propagation rules [70].

For optimal normalization of data, we evaluated the stable gene expression of four references in our dataset. References (*Ca4g6410, PP2A, PPR* and *EF1a*) were chosen based on previous reports that had showed high stability values across experiments [49,71,72]. To evaluate the stability of the reference genes, we used the geNorm algorithm [73]. The pilot study indicated that *Ca4g6410* and *PP2A* were the most stable references with stability values M = 0.23. PCR efficiency (E) of the references was, respectively: E = 1.98 ± 0.05 and E = 1.98 ± 0.04 (mean ± SD).

## 4. Conclusions

Plants are continuously exposed to different types of abiotic and biotic stresses. Plant molecular responses induce the generation of reactive oxygen species, which in turn interfere with cell structure and metabolic balance in cells. To protect themselves, plants produce stress-responsive proteins, such as ALDHs, which contribute to aldehyde homeostasis as scavengers to eliminate toxic aldehydes. In the present study, performing a series of comprehensive analyses including chickpea genome analysis, ALDH genes identification and naming, comparative phylogeny and ALDH genes expression profiles assessment, we identified 27 unique ALDH sequences in the chickpea genome. Most of the sequences originated from duplication events. Chickpea exhibits a remarkable expansion in the ALDH3 family, showing one of the largest numbers of members compared to other plant species. The expression results give consistent support in the functional roles of the ALDH genes, mostly being involved in responses to desiccation and drought conditions, but also responses to biotic stress. Furthermore, the expression data revealed that some of the duplicated members in a group exhibited different expression patterns, suggesting that functional diversification is a feature in the evolution of these genes. Based on expression data support and close phylogenetic relationships with other well-characterized proteins, some chickpea ALDHs (such as LOC101511819 or LOC101510937) are good candidates for further characterization. These candidates may become targets for improving chickpea adaptation to adverse environmental or biotic stresses in breeding programs. Furthermore, our study also provides a foundation for further comparative genomic analyses and a framework to trace the dynamic evolution of the ALDH superfamily.

## Figures and Tables

**Table 1:** The aldehyde dehydrogenase gene superfamily in chickpea.

Gene ID	Locus ID	Chr	Chr Start	Chr End	Strand	RNA ID	Exons	Protein ID	Protein Length (aa)	Molecular Weight	Isoforms Number	Isoelectric Point
CaALDH3F1	LOC101497113	Ca1	11,135,019	11,130,555	-	XM_004486911	10	XP_004486968	494	54.82	1	8.10
CaALDH22A1	LOC101512347	Ca1	17,180,053	17,171,549	-	XM_004487701	14	XP_004487758	595	65.35	1	6.72
CaALDH7A1	LOC101513733	Ca1	23,039,421	23,046,624	+	XM_012718791	15	XP_012574245	508	54.09	2	5.70
CaALDH5F1	LOC101506901	Ca1	37,645,851	37,658,363	+	XM_004488493	20	XP_004488550	530	56.59	1	6.58
CaALDH18B3	LOC101499756	Ca3	8,714,224	8,700,487	-	XM_012713409	20	XP_012568863	717	77.75	2	5.96
CaALDH3H3	LOC101515558	Ca4	38,313,842	38,325,387	+	XM_004498289	10	XP_004498346	488	53.06	1	8.43
CaALDH10A8	LOC101507930	Ca5	39,963,842	39,971,506	+	XM_004501904	15	XP_004501961	503	54.53	1	5.37
CaALDH3H2	LOC101510937	Ca5	44,008,221	44,002,223	-	XM_004502425	11	XP_004502482	488	53.18	3	7.01
CaALDH3H4	LOC101511680	Ca5	44,024,284	44,016,817	-	XM_004502428	10	XP_004502485	486	52.99	1	8.33
CaALDH18B2	LOC101490622	Ca6	1,317,458	1,311,762	-	XM_012716567	21	XP_012572021	715	77.65	1	6.62
CaALDH2C5	LOC101493969	Ca6	3,278,797	3,283,156	+	XM_004503375	10	XP_004503432	480	52.33	1	6.44
CaALDH3H1	LOC101505038	Ca6	6,829,385	6,835,166	+	XM_004503842	12	XP_004503899	542	59.76	2	7.96
CaALDH6B2	LOC101490310	Ca6	15,177,302	15,170,648	-	XM_004504810	19	XP_004504867	539	57.63	1	7.08
CaALDH18B1	LOC101512568	Ca6	44,541,903	44,527,947	-	XM_027335197	21	XP_027190998	759	82.38	4	6.82
CaALDH3F2	LOC101491914	Ca6	53,416,538	53,426,529	+	XM_004507038	10	XP_004507095	488	54.56	1	9.22
CaALDH11A3	LOC101510843	Ca7	1,260,647	1,264,733	+	XM_004507665	9	XP_004507722	496	52.81	1	6.53
CaALDH12A1	LOC101490107	Ca7	8,738,308	8,744,740	+	XM_004508712	16	XP_004508769	553	61.30	1	6.17
CaALDH10A9	LOC101506136	Ca7	9,155,132	9,150,438	-	XM_004508765	14	XP_004508822	503	54.40	1	5.37
CaALDH2B4	LOC101490532	Ca7	9,459,504	9,464,830	+	XM_004508796	12	XP_004508853	536	58.58	3	7.57
CaALDH3F3	LOC101511819	Ca7	14,455,263	14,450,584	-	XM_012718277	10	XP_012573731	488	54.13	1	7.99
CaALDH2B7	LOC101492709	Ca7	21,404,965	21,399,791	-	XM_004509777	11	XP_004509834	539	58.04	1	6.58
CaALDH2C6	LOC101513875	Ca8	14,992,271	14,983,690	-	XM_012719313	9	XP_012574767	498	44.10	1	5.55
CaALDH2C4	LOC101514219	Ca8	14,998,332	15,002,867	+	XM_004512910	9	XP_004512967	503	54.64	2	6.19
CaALDH3H7	LOC101502106	Un	0	0	-	XM_004514027	0	XP_004514084	134	15.07	1	9.49
CaALDH18B4	LOC105852801	Un	0	0	-	XM_012719507	0	XP_012574961	248	27.72	1	4.34
CaALDH3H5	LOC101497514	Un	0	0	-	XM_027330984	0	XP_027186785	214	23.51	4	9.21
CaALDH3H6	LOC101488602	Un	0	0	-	XM_027330333	0	XP_027186134	145	16.19	4	9.47

## Data Availability

All data generated or analyzed during this study are included in this article and its Supplementary Information Files.

## Supplementary Materials

The following are available online at https://www.mdpi.com/article/10.3390/plants10112429/s1, Figure S1: Gene duplications. Similarity of ALDH genes. Red color shows the highest similarity (>95% identity), followed by orange (90–95%) and green (80–90%) colors. Figure S2: Gene expression levels of selected ALDH genes in response to *Fusarium oxysporum*. Table S1: Identification of PF00171, PS00687, PS00070 and predicted location of ALDH proteins in chickpea. Table S2: Synteny blocks of aldehyde dehydrogenase (ALDH) genes between chickpea and Medicago truncatula genomes. Table S3: Tissue and stress distribution profile of chickpea ALDH genes based on number of expressed sequence tags (ESTs) present in the NCBI’s EST Database. Table S4: Primers for qPCR. PCR efficiencies € calculated according to the equation (1 + E) = 10^slope^. Table S5: Differentially regulated ALDH genes. RNA-seq analysis was conducted on two contrasting genotypes in their response to drought stress.
