# Supplementary figures and images for: Aldehyde Dehydrogenase 3 Is an Expanded Gene Family with Potential Adaptive Roles in Chickpea
Source: Plants (Basel). 2021 Nov 10;10(11):2429. doi: 10.3390/plants10112429 (PMC8619295; doi:10.3390/plants10112429)

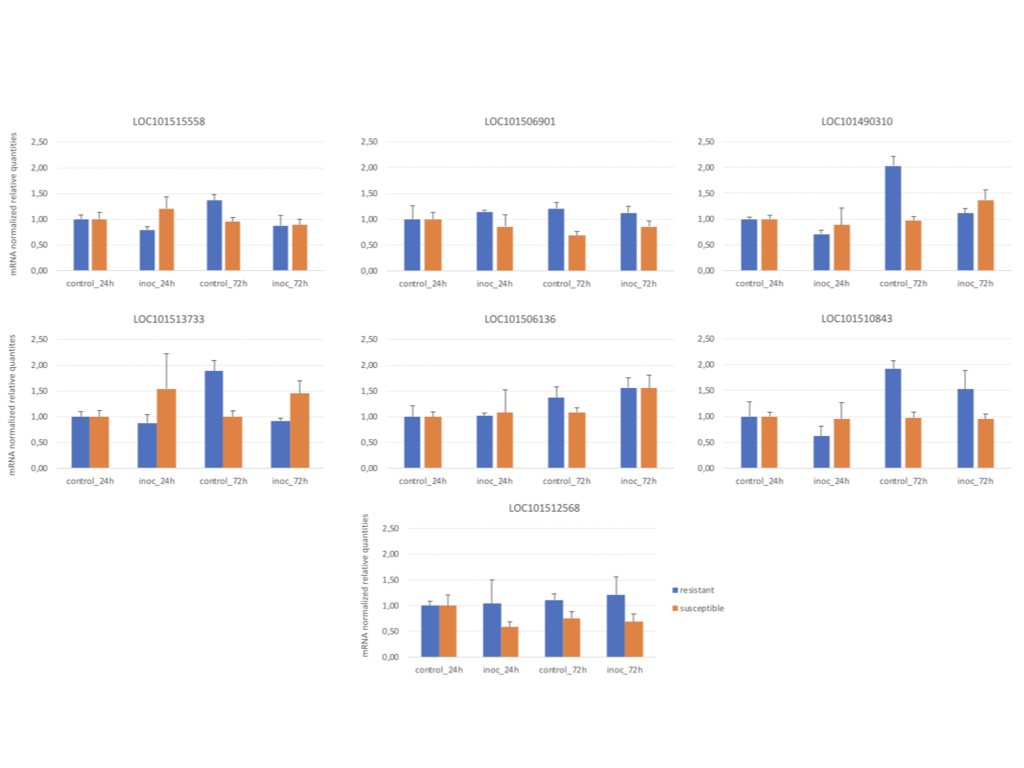

Supplement: Supplementary file 1 [file plants-10-02429-s001.zip › SuppFiles/Fig S2.tiff]

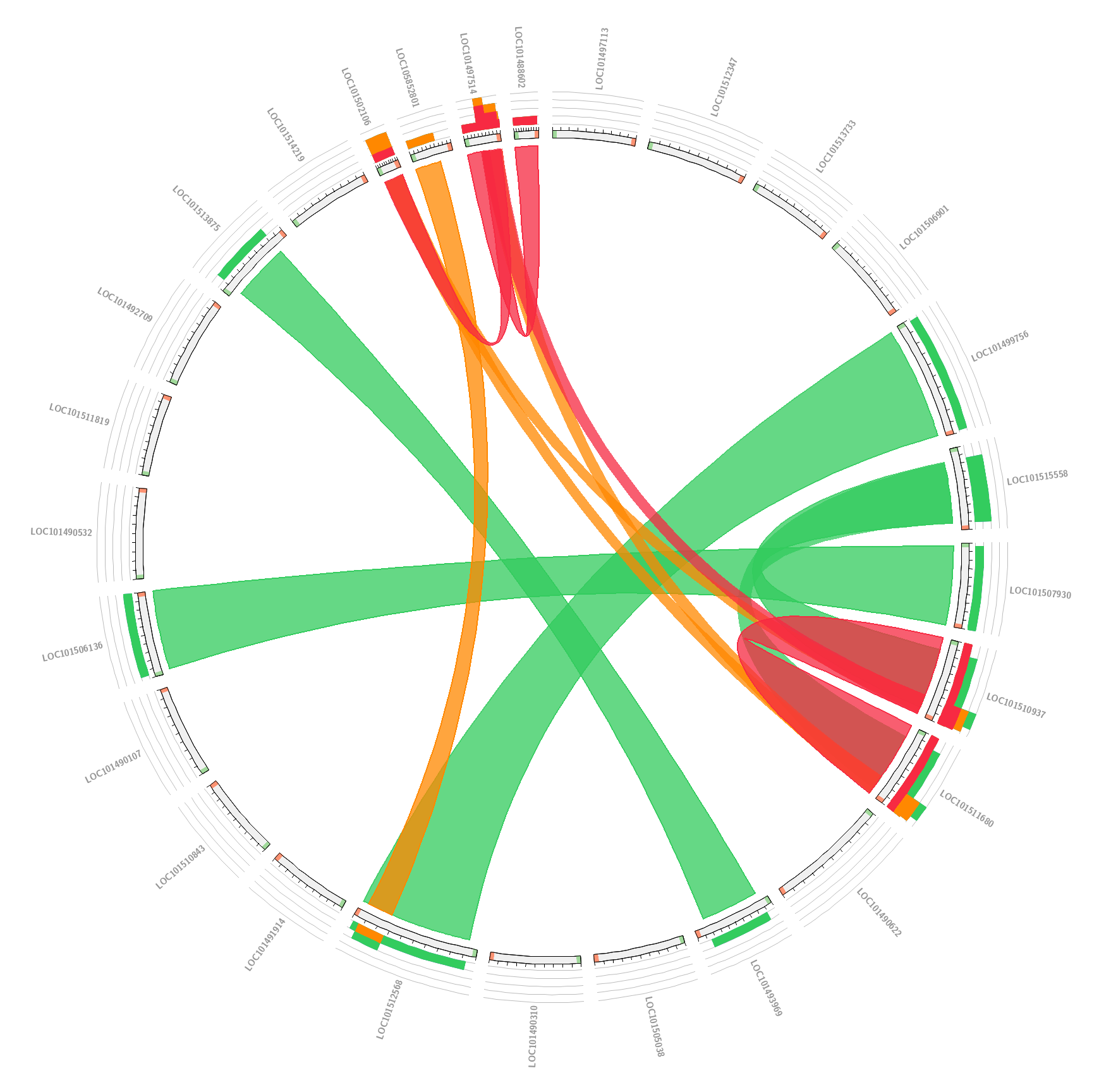

Supplement: Supplementary file 1 [file plants-10-02429-s001.zip › SuppFiles/FigureS1.tiff]
